# Comparison of USG-guided or landmark approach fascia iliaca compartment block for positioning in elderly hip fracture patients with spinal anesthesia: a randomized controlled observational study

**DOI:** 10.3906/sag-2011-254

**Published:** 2021-07-04

**Authors:** Tuna ERTÜRK, İbrahim GÜNDOĞMUŞ, Temel GÜNER, Cengiz YILDIRIM, Ayşın ERSOY

**Affiliations:** 1 Department of Anesthesiology, University of Health Sciences, Sultan 2. Abdülhamid Han Training and Research Hospital, İstanbul, Turkey; 2 Department of Psychiatry, University of Health Sciences, Kırıkkale Yüksek İhtisas Hospital, Kırıkkale, Turkey; 3 Department of Orthopedics and Traumatology, University of Health Sciences, Sultan 2. Abdülhamid Han Training and Research Hospital, İstanbul, Turkey

**Keywords:** Fascia iliaca compartment block, hip fractures, spinal anesthesia, position pain, ultrasonography

## Abstract

**Background/aim:**

Currently, the elderly population in the world is rapidly increasing due to technological developments and convenient access to health services. Due to comorbidities in elderly patients, hip fractures are frequently observed after exposure to environmental trauma. To reduce pain during positioning in spinal anesthesia, fascia iliaca compartment block (FICB) can be applied easily and reliably.

In our study, we aimed to compare the analgesic effects and duration of fascia iliaca compartment blocks performed with USG guidance or the landmark approach methods for relieving spinal anesthesia position pain.

**Materials and methods:**

Our study included 100 patients undergoing operations due to hip fracture and administered spinal anesthesia after FICB. The group with USG-guided FICB (USG) had the blockage needle advanced to the compartment under the fascia iliaca, and 15 mL bupivacaine + 10 mL 2% lidocaine was administered. They were placed in sitting position for spinal anesthesia 20 min later and procedure duration and numerical rating scale (NRS) scores were recorded. In the group with landmark approach FICB (LAND), the spina iliaca anterior superior (SIAS) and pubic tubercle were connected with a line. The same amount of local anesthetic was administered to the external 1/3 portion of this line with the double pop technique. Procedure duration and NRS scores were recorded.

**Results:**

There was no statistically significant difference between the two groups in terms of NRS scores (p: 0.073). There was a statistically significant difference in duration of FICB administration between the two groups (p < 0.001).

**Conclusion:**

Both USG-guided and landmark approach FICB methods provide adequate and similar analgesia for positioning in spinal anesthesia. However, in cases where there is no problem with access to the ultrasound device or time, safer blockage can be provided by imaging neurovascular structures with ultrasound.

## 1. Introduction

Hip fractures linked to causes like trauma and/or falls affect nearly 1.6 million people in the world in general. Due to the numerical increase in the geriatric population, it is thought that this rate will rapidly increase within the next 30 years [1]. In 2009, 24 thousand hip fractures were reported in Turkey, while it is estimated that in 2035 this number will reach 64 thousand per year [2].

Systemic diseases, decreased reflexes, and cerebrovascular events in the elderly patient group expose these patients to more environmental trauma and cause more hip fractures in this population. In addition, reduced bone fusion in this age group is another reason that increases the incidence of fracture development [3]. In the elderly, hip fracture is the most commonly observed fracture type after distal radius fracture. Of these fractures, 90% are observed in patients over 65 years of age.

The anesthetic approach in hip fractures is linked to the patient’s hemodynamics, physiological status and comorbidities. General anesthesia represents a risk in patients with severe respiratory disorders. Regional anesthesia is chosen considering advantages like reduced thromboembolism risk, less blood loss, reduced cognitive disorders, and shorter hospitalization [4]. However, spinal anesthesia is avoided due to pain in the fracture site during spinal anesthesia. To reduce pain occurring during the positioning stage for hip fractures, it is necessary to block the femoral and lateral femoral cutaneous branches of the lumbar plexus and if required the obturator nerves.

Psoas compartment block (PCB), lumbar plexus block (LPB), fascia iliaca compartment block (FICB), and femoral nerve block (FNB) are among blocks with analgesic efficacy after total hip arthroplasty [5]. They are also used to resolve positioning pain in hip fracture surgeries. FICB can be easily applied with USG guidance or the landmark approach method.

FICB is applied more easily and safely than other blockage methods because the intervention area is far from the neurovascular structures [6]. Specifically, the femoral, lateral femoral cutaneous, and obturator nerves can be blocked with local anesthesia (LA) injected under the fascia of the iliac muscle [7].

The primary aim in our study is to compare the analgesic effects and duration of fascia iliaca compartment blocks performed with USG-guidance or Landmark approach methods to relieve spinal anesthesia position pain due to hip fractures. The secondary aim of our study is to relieve spinal anesthesia position pain in elderly patients and to perform spinal anesthesia more easily and successfully.

## 2. Materials and methods

This single-center, prospective observational clinical study included 100 patients undergoing surgery due to hip fracture under spinal anesthesia after FICB administration, in the American Society of Anesthesiologists (ASA) physical status classification I-II-III (ASA, ASA II, ASA III) and 65–90-year-old patient group. Standardized Mini Mental Test (SMMT) was applied to all patients. Written informed consent was obtained from each patient. Cases were randomly divided into 2 groups: USG-guided FICB (USG) (n = 50) or landmark approach FICB (LAND) (n = 50).

Exclusion criteria for the study were age younger than 65 years or older than 90 years, ASA physical status classification IV, contraindications for block administered to the inguinal region and spinal anesthesia, lack of consent by themselves or legal heirs, lack of cooperation- orientation, peripheral neuropathy, known allergy to amid-type local anesthetics, bleeding diathesis, moderate or severe kidney and liver function disorder, and not accepting FICB administration.

Demographic data were recorded during the preoperative assessment. None of the patients in the study had a SMMT score below 23; therefore, no patient was excluded from the study.

**Figure 1:**
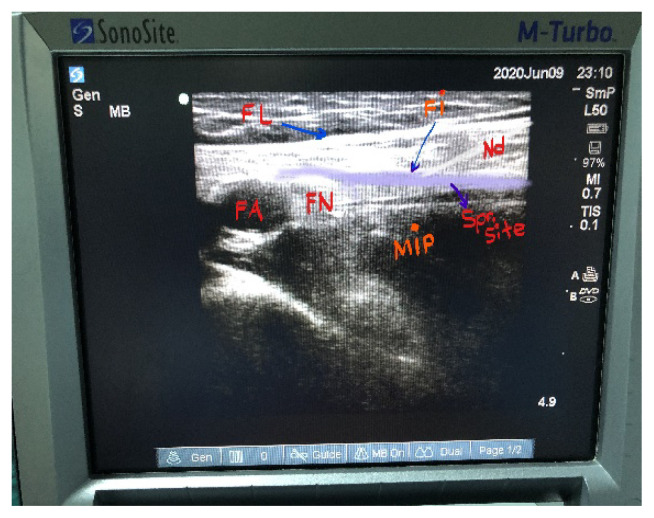
USG guided FICB.

In the FICB (USG) group, after sterilizing the procedure region, the USG probe was covered for sterility and then the fascia iliaca was imaged (Figure 1). After subcutaneous 2 mL 2% prilocaine application, the 22G 50 mm block needle was advanced to the compartment under fascia iliaca, and 25 mL of local anesthetic (15 mL 0.5% bupivacaine [Marcaine vial, Astra Zeneca İlaç, İstanbul] + 10 mL 2% lidocaine [Aritmal amp, Osel İlaç, İstanbul]) was administered to this area. The duration was recorded from the start of the imaging process to the removal of the block needle. After waiting 20 min, sensorial block was assessed by cold application to the anterior (femoral nerve), medial (obturator nerve), and lateral (lateral femoral cutaneous nerve) faces of the two thighs. NRS scores were recorded during the period in sitting position for spinal anesthesia.

**Figure 2:**
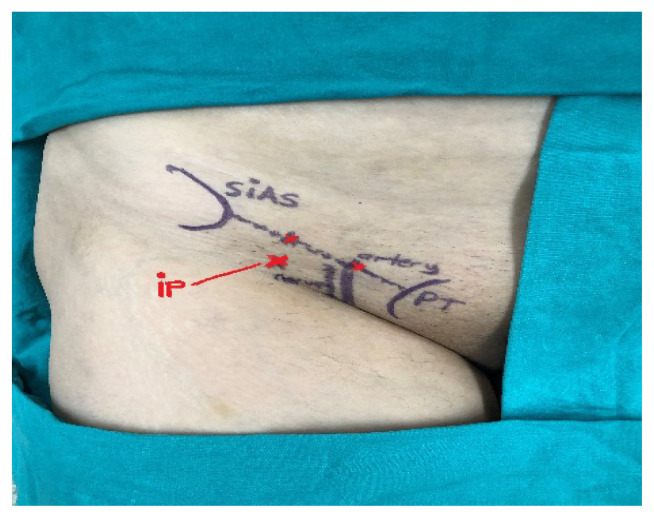
Landmark approach FICB. FL: Fascia lata, FI: Fascia iliaca, Nd: Needle FN: Femoral nerve SIAS: Spina iliaca anterior superior, PT: Pubic tubercle FA: Femoral artery, MIP: Musculus iliopsoas: Spr. Site: Spreading site IP: Injection point

In the FICB (LAND) group, after sterilizing the procedure region, a line was drawn from SIAS to the pubic tubercle on the same side. The line was divided into three equal parts and the join between the middle and external 1/3 sections was marked, and an entry point 2 cm below this point was determined (Figure 2). In this region, after administration of 2 mL 2% prilocaine (Citanest^©^) skin-subdermal, subdermal entry was performed with a 22 G 50 mm block needle. When advancing the needle, a pop sensation was felt 2 times due to resistance loss on passing the fascia lata and fascia iliaca, and negative aspiration was performed. Then 25 mL of local anesthetic (15 mL 0.5% bupivacaine [Marcaine vial, Astra Zeneca İlaç, Istanbul] + 10 mL 2% lidocaine [Aritmal amp, Osel İlaç, İstanbul]) was administered to this area. The duration was recorded from the beginning of the anatomic marking procedure to the removal of the block needle. Similarly, sensory block was assessed after 20 min, and the NRS scores were recorded during the period in sitting position for spinal anesthesia.

After these procedures, spinal anesthesia was administered to the patients with 15 mL 0.5% hyperbaric bupivacaine (Marcaine^®^ spinal heavy, Astra Zeneca) at the L3–L4 level. After development of sensory nerve block reaching the T10 dermatome, appropriate position for surgery was given. At the end of the surgery, the patients were transferred to the postoperative care unit. Patients with class 0–1 on the Bromage scale and Aldrete score 9–10 were transferred to the orthopedic inpatient service.

All FICB and spinal anesthesia procedures were performed by the same anesthesiologist who had previously performed FICB in at least 10 patients in both groups.

### 2.1. Statistical analysis

Mean, standard deviation, median, minimum, maximum, frequency, and percentage values were used for descriptive statistics. The distribution of variables was measured with the Kolmogorov–Smirnov test. Independent samples t-test and the Mann–Whitney U test were used for quantitative independent data analysis. The Wilcoxon test was used for dependent quantitative data analysis. The chi-squared test was used for qualitative independent data analysis. The analyses were performed using SPSS 26.0 program.

For the power analysis in our study, the calculation was made according to a webpage^1^. Power analysis was performed within 80% confidence interval and the number of patients in each group was determined as 50 patients, with reference to Kacha et al.’s study [22].

## 3. Results

The study was completed with a total of 100 patients, of whom 51 were women and 49 were men. When demographic data, ASA class distribution (p: 0.771) and SMMT results (0.427) are compared, there was no statistically significant difference between the LAND and USG groups.

The median NRS scores were recorded in both groups and there was no statistically significant difference between the groups (p: 0.073). Additionally, the FICB administration duration was median 174 s in the USG group and 72 s in the LAND group and there was a statistically significant difference between the two groups (p < 0.001) (Table 1).

**Figure 3:**
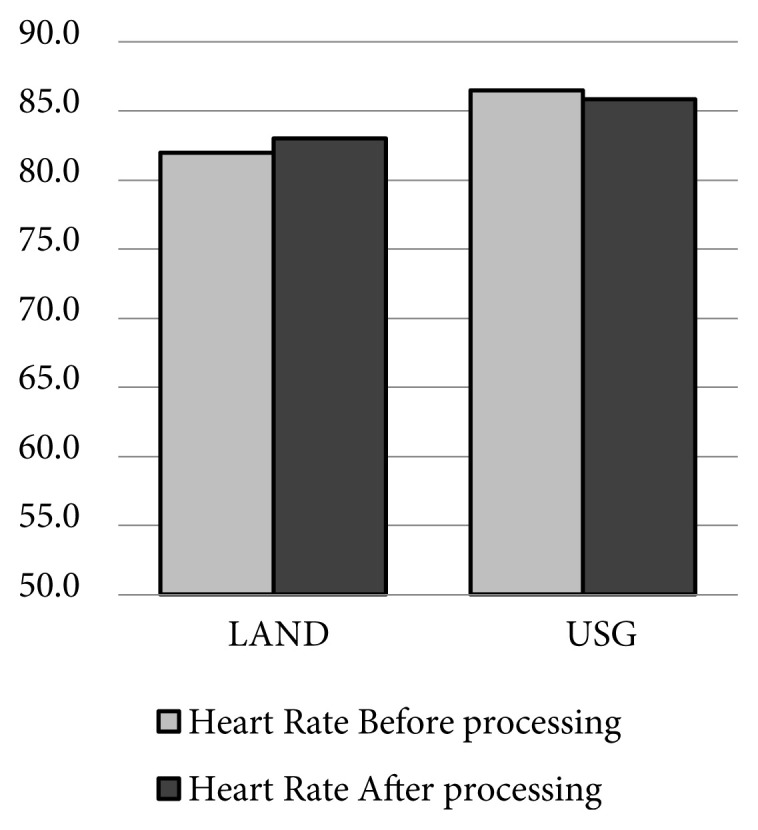
HR and MAP variations before and after positioning.

When the LAND and USG groups were compared during the procedure, heart rate (HR) values (p: 0.182), systolic–diastolic and mean arterial pressure (MAP) (p: 0.191) did not show significant differences (Table 2; Figure 3). As a result, the variation in pain-supportive hemodynamic parameters before and after the positioning procedure was not different.

## 4. Discussion

In this study, we aimed to compare the analgesic effects of fascia iliaca compartment block performed with the USG-guided method or landmark approach method for relieving spinal anesthesia position pain. In addition, we planned to compare the block duration administered with both methods. We found that the analgesic effects of FICB applied with both methods were similar and sufficient to relieve positional pain during spinal anesthesia. FICB administration duration was longer in the USG group.

The SMMT test, which is used for evaluation of neurocognitive function, was applied to all our patients to evaluate the accuracy of NRS scores [8]. Neurocognitive deficiency was not observed in any of our patients.

Surgeries like trauma-linked hip fracture repair and hip prosthesis are frequently performed in geriatric patients. The reduction of physiological adaptation capacities and presence of comorbid systemic diseases in geriatric patients increase the complication risks that may occur during and after the operation. Regional anesthesia is preferred in elderly patients to reduce complications, intensive care requirement, duration of hospitalization, and morbidity–mortality rates [9–13]. Advantages such as minimal drug cost, prevention of surgery-related immunosuppression, reduction in postoperative thromboembolism risk, reduction in blood loss, reduction in postoperative confusion incidence, and rapid patient turnover make neuraxial anesthesia a preferred method for many surgical procedures [11,14]. In our study, the mean age was 76.5 years in the LAND group and 75 years in the USG group. We chose regional anesthesia for the surgical treatment of hip fractures for reasons such as low mortality and morbidity. We applied FICB for positional pain relief for spinal anesthesia because it does not require much experience and is a safe block away from neurovascular structures.

A study by Chow et al. [15] stated that postoperative delirium development rates were lower for patients undergoing surgery with regional anesthesia compared to general anesthesia. The lower observation of delirium in patients with regional anesthesia also reduces postoperative cognitive dysfunction and mortality. In our study, we observed only 5 patients (5%) in our intensive care unit for a short time postoperatively due to comorbidities. FICB provided effective postoperative analgesia in all our patients. Therefore, postoperative delirium was not observed in any of our patients.

FICB was first described in 1989 and was performed initially on children and later on adults. It was mainly used to provide analgesia following surgical procedures in the hip, femur and knee, treatment of burns on the thigh and in prehospital treatment of fracture femur [16,17]. FICB is extremely effective in blocking the femoral nerve and lateral femoral cutaneous nerve [18]. FICB can be applied easily. In addition, the risk of complications is low since it is administered away from neurovascular structures. Although FICB has been described very recently, there is a broad field of use because it is a block that can be applied easily and in a short duration, with low cost and without requiring serious experience [19]. In our study, we did not have any application that involved difficulties or complications. Due to these advantages, we think that FICB can be applied safely in emergency services and orthopedic services.

The mechanism of this block is blockage of the femoral, lateral femoral cutaneous, and obturator nerves under the fascia iliaca. Sufficient amounts of local anesthetic administered under the fascia iliaca induces block in the compartment under the fascia, even if it spreads somewhat distant from the nerves [20]. In our study, we think that the reason for the anatomic landmark approach FICB block having a similar analgesic effect with USG-guided block is subfascial extension.

Kumar et al. [21] used FICB for pain occurring linked to position during hip fracture surgeries and found that 86% of patients had good results on their assessment of patient satisfaction. Similarly, there are many studies showing that FICB is effective in relieving spinal anesthesia position pain in hip replacement and femur fracture surgeries [22–24]. In our study, we observed a high level of patient satisfaction with the block we administered with both methods. The median values for NRS scores in both groups were 2 and positioning pain before spinal anesthesia was significantly resolved.

A metaanalysis reported that USG-guided regional anesthesia had higher success rates to a clinically significant degree compared to the landmark technique and that analgesia could be obtained with more rapid onset, long-duration block, and lower vascular puncture risk [25]. In another study, USG-guided and anatomical landmark approach methods were compared, and USG-guided FICB provided significantly more effective sensory and motor blockade [26]. In our study, it was observed that two patients in the LAND group had high NRS scores. However, we could not find any statistical difference between the groups in terms of analgesic effect, and we could not find any findings suggestive of vascular-neuronal injection.

## 5. Conclusion

The point we want to emphasize in our study is that although different blocking methods such as PCB, LPB, and FNB are used to relieve spinal anesthesia position pain in hip fractures, FICB can be applied more easily and safely than other blocking methods in the region away from neurovascular structures. Because of these advantages, FICB is preferred more frequently than other blocking methods in emergency services and preoperative orthopedic services, and patient comfort is increased by reducing the pain of the patients.

Although the success of USG-guided FICB has come to the fore in other studies, in our study, equal and adequate analgesia was provided with the anatomical landmark method and USG-guided blockade.

In conclusion, FICB provides adequate and similar analgesic levels for positioning in spinal anesthesia when applied with both USG-guided and landmark approach methods. USG-guided FICB has the disadvantage of requiring a device and a long duration for administration. Since the operation area is far from neurovascular structures, it may not require imaging with ultrasound. However, imaging of all neurovascular structures with ultrasound will provide more reliable blockage. In conditions where there is an ultrasound device and time is not limited, the procedure should be performed with USG guidance.

In cases where there is no ultrasound device, the landmark approach FICB method provides sufficient analgesic effect. FICB applied by both methods appears to be reliable and easy to administer to relieve positioning pain for all hip fracture patients undergoing spinal anesthesia.

## Figures and Tables

**Table 1:** Comparison of demographic data, ASA class, and NRS scores.

		LAND				USG				p	
		Mean ± SD	n	%	Median	Mean ± SD	n	-%	Median		
Age		75.2 ± 9.9			76.5	74.5 ± 9.9			75.0	0.702	t
Sex	Female		24	48%			27	54%		0.548	X²
	Male		26	52%			23	46%			
PİCU			3	6%			2	4%			
Height		166.6 ± 7.8			168.0	165.8 ± 7.6			166.0	0.519	m
Weight		75.6 ± 8.0			76.0	77.0 ± 8.1			78.0	0.272	m
BMI		27.3 ± 2.9			27.0	28.2 ± 3.6			28.0	0.258	m
ASA	I		7	14%			5	10%		0.771	X²
	II		28	56%			31	62%			
	III		15	30%			14	28%			
SpO_2_		95.9 ± 2.0			96.0	95.6 ± 1.9			96.0	0.300	m
SMMT		28.1 ± 1.4			28.0	28.4 ± 1.4			29.0	0.427	m
Procedure time		78.5 ± 19.1			72.0	179.4 ± 21.8			174.0	**<0.001**	m
NRS Scores		2.6 ± 1.7			2.0	2.2 ± 1.5			2.0	0.073	m

m The Mann–Whitney U test /

X² chi-squared test /

T Independent samples t-test

**Table 2:** Heart rate, systolic–diastolic and mean arterial pressure.

	LAND		USG			
	Mean ± SD	Median	Mean ± SD	Median	p	
***Heart Rate***						
Before procedure(BP)	82.0 ± 10.4	81.5	86.5 ± 11.7	85.0	0.056	^m^
After procedure(AP)	83.0 ± 11.7	82.5	85.8 ± 13.0	85.0	0.302	^m^
BP/AP Variation	1.00 ± 6.84	1.00	−1.04 ± 7.22	−2.00	0.182	^m^
Variation in Group P	0.646 ^w^		0.185 ^w^			
***Systolic pressure***						
Before procedure(BP)	133.5 ± 16.1	129.5	131.9 ± 12.6	130.0	0.915	^m^
After procedure(AP)	134.2 ± 14.1	133.0	130.6 ± 12.0	128.5	0.177	^m^
BP/AP Variation	0.68 ± 13.48	1.50	−1.32 ± 9.31	−3.0	0.187	^m^
Variation in Group P	0.650 ^w^		0.160 ^w^			
***Diastolic pressure***						
Before procedure(BP)	82.5 ± 10.0	83.5	82.7 ± 8.6	84.0	0.844	^m^
After procedure(AP)	83.5 ± 9.4	82.0	80.3 ± 8.5	81.0	0.105	^m^
BP/AP Variation	0.96 ± 6.29	−0.50	−2.46 ± 6.45	−3.0	**0.012**	^m^
Variation in Group P	0.526 ^w^		**0.005** ^w^			
***Mean Arterial Pressure***						
Before procedure(BP)	99.6 ± 11.8	98.5	99.2 ± 9.7	98.0	0.863	^m^
After procedure(AP)	99.5 ± 10.6	97.0	97.1 ± 8.4	96.0	0.227	^m^
BP/AP Variation	−0.08 ± 8.83	−2.00	−2.06 ± 6.86	−3.00	0.191	^m^
Variation in Group P	0.825 ^w^		0.052 ^w^			

## Footnotes

1
https://www.dssresearch.com/KnowledgeCenter/toolkitcalculators/samplesizecalculators.aspx
